# Dyadic Adjustment and Spiritual Activities in Parents of Children with Cystic Fibrosis

**DOI:** 10.3390/rel5020385

**Published:** 2014-04-11

**Authors:** Daniel H. Grossoehme, Rhonda Szczesniak, Caitlin Dodd, Lisa Opipari-Arrigan

**Affiliations:** 1 Division of Pulmonary Medicine, Cincinnati Children’s Hospital Medical Center, MLC2021, 3333 Burnet Avenue, Cincinnati, OH 45229, USA; 2 Division of Biostatistics and Epidemiology, Cincinnati Children’s Hospital Medical Center, 3333 Burnet Avenue, Cincinnati, OH 45229, USA; 3 Medical Informatics Department, Erasmus University, Rotterdam 3015 GE, The Netherlands; 4 Division of Behavioral Medicine and Clinical Psychology, Cincinnati Children’s Hospital Medical Center, 3333 Burnet Avenue, Cincinnati, OH 45229, USA

**Keywords:** marital adjustment, spirituality, cystic fibrosis, parent

## Abstract

Children’s diseases can negatively impact marital adjustment and contribute to poorer child health outcomes. To cope with increased marital stress and childhood diseases severity, many people turn to spirituality. While most studies show a positive relationship between spirituality and marital adjustment, spirituality has typically been measured only in terms of individual behaviors. Using the Dyadic Adjustment Scale (DAS) and Daily Phone Diary data from a sample of 126 parents of children with cystic fibrosis as a context for increased marital stress, spiritual behavior of mother-father dyads and of whole families were used as predictors of marital adjustment. Frequency and duration of individual, dyadic and familial spiritual activities correlated positively with dyadic adjustment. Significant differences in spiritual activities existed between couples with marital adjustment scores above and below the cutoff for distress. The only significant factors in regressions of spiritual activities on marital adjustment scores were number of pulmonary exacerbations and parent age. Higher odds of maintaining a marital adjustment score greater than 100 were significantly associated with spending approximately twelve minutes per day in individual, but not conjugal or familial, spiritual activities. The Daily Phone Diary is a feasible tool to study conjugal and familial activities and their relationships with beliefs and attitudes, including spirituality.

## 1. Introduction

Children’s problems can add strain to a marriage, whether due to an infant’s crying or due to the severity of a child’s chronic diseases, yet there is wide variation in how such problems impact marriages and families [1,2] Simply having a child with a disability or chronic illness does not by itself predict poor marital adjustment. Understanding how different aspects of the family environment impact marital adjustment in the face of chronic stressors, such as childhood disability or chronic illness, is critical for helping families cope with distress and for bolstering their resilience.

Cystic fibrosis (CF) is the most common genetic disease among Caucasian and affects approximately 30,000 children and adults in the US and approximately 70,000 worldwide [3]. The median predicted survival age in 2009 was mid-30s [3]. The genetic mutation causing CF disrupts the normal intracellular transport of sodium ions, which results in the cells’ inability to clear mucus. A buildup of mucus in the lung tissue frequently results in a bacterial infection and decreased lung function. Mucus buildup also prevents proper absorption through the intestinal walls. Nutrition is further affected for many people by having a non-functioning pancreas, which necessitates taking supplemental enzymes to digest food properly. CF may also cause liver failure, sinusitis, and or diabetes. In order to counteract the progressive nature of the disease, some form of chest physiotherapy (airway clearance) is usually performed, typically twice a day for approximately 30 minutes. In addition, the nebulized hypertonic saline may be prescribed to loosen mucus before airway clearance, and or nebulized antibiotics to fight bacterial infections. This daily treatment regimen is burdensome, requiring 90 to 120 minutes on a typical day; the number of airway clearance treatments is frequently increased to four times daily if a child feels they are becoming sick.

**Figure 1:**
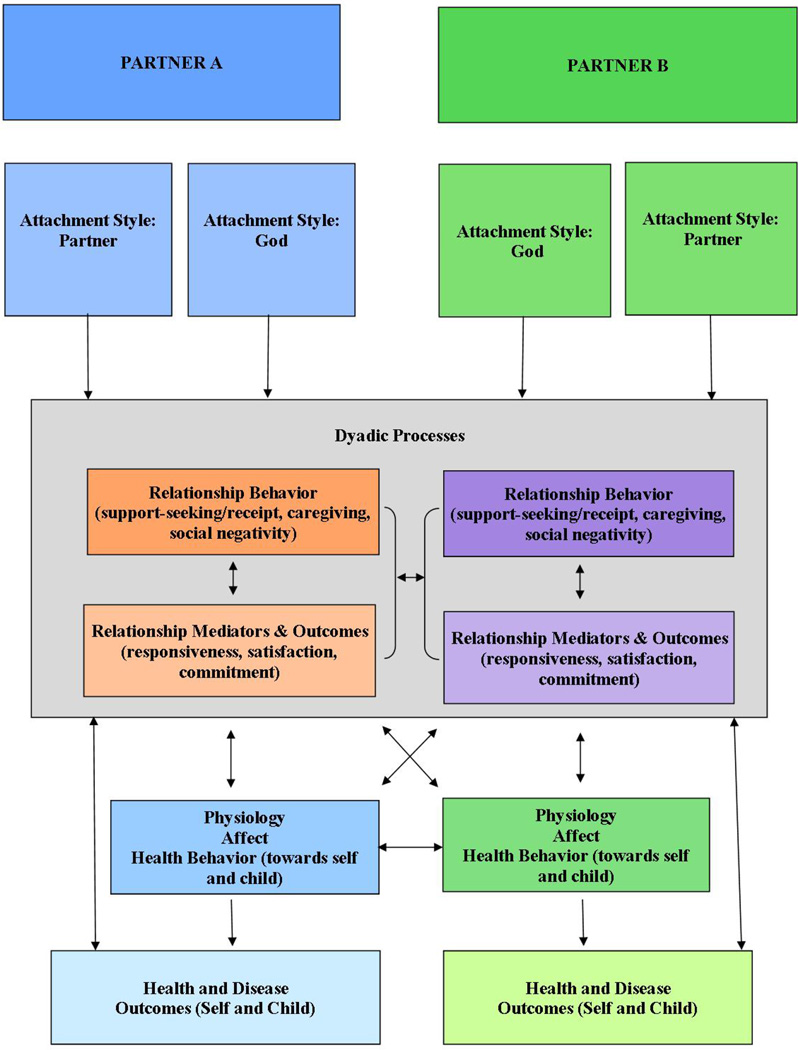
Adapted conceptual model of dyadic process, attachment, and health behaviors.

A child’s chronic illness, especially one that is potentially life-shortening, affects a couple’s and a family’s relationship, and may lead to changed behaviors [4]. Exploring dyadic constructs that relate to health may be more informative than studying individuals. Attachment Theory offers a conceptual model through which to view the relationships between dyadic constructs and child health. An adaptation of the theoretical model proposed by Pietromonaco and colleagues [5] provides a useful framework for understanding the relationship between dyadic adjustment and child health (see Figure 1). Their model extends attachment theory beyond its usual parent-child focus to focusing on distressed adults who relate to their spouse or partner as an “attachment figure” to lower their distress. Attachment theory has also been applied to adults’ relationship with God [6]. For some couples, God provides a secure base for both their personal development as well as enabling them to overcome some dyadic dysfunction [4].

Religion and spirituality are aspects of the family environment that may contribute to coping with the impact that chronic stress has on a marriage. They provide meaning to events and offer behavioral guidance, comfort, a sense of connectedness and ritual. Religion and spirituality are strong influences on how many individuals independently cope with stress [7–9] and have been found to be important coping strategies for most parents of children with chronic disorders [10–12]. While previous research has examined the relationship between religion, spirituality and marital adjustment, less is known about these relationships in the context of chronic stress. Koenig and colleagues have reviewed this literature [13]. They discuss several studies in which increased religiosity was associated with increased marital adjustment. Beliefs and practices alone do not appear to be significant predictors; Koenig and colleagues suggest that it is the interiorization of beliefs or practices in a partnered individual which is significant for one’s marital adjustment rather than the outward expression of beliefs or practices. Mahoney’s review of a decade of research in the area of spirituality and family life included studies showing religion or spirituality as either inversely related, or unrelated, to marital conflict [14]. Spirituality has been shown to positively relate to marital adjustment [15] and spiritual growth and activities have been named as a “staple” in some relationships [16]. The internalization of the beliefs for which spiritual activities are observable manifestations may be the most important factor [17].

Previous research in this area is limited by a focus on measuring spirituality in terms of one or two general questions and only in terms of an individual’s personal spiritual behaviors. Spirituality is a multi-faceted construct that cannot adequately be measured by single items but rather is best conceptualized in terms of spiritual beliefs, practices and community [18]. Using a novel approach for this field of inquiry (daily, 24-hour recalls that assess all spiritual behaviors engaged in by the individual, couple, and family), and using cystic fibrosis (CF) as a context that places stress on a marriage, this analysis was developed to address the following hypotheses. First, that the frequency and duration of individual, conjugal and familial spiritual activities would be positively correlated with marital adjustment; second, that significant differences would exist in the frequency and duration of spiritual activities for individuals with Dyadic Adjustment Scale (DAS) scores above and below the clinical cutoff for distress; third, that frequency and duration of conjugal and familial spiritual activities would be positively associated with marital adjustment in regression models after controlling for disease severity; and fourth, that a minimal duration of spiritual activities per day could be determined below which marital adjustment would be considered more likely distressed.

## 2. Methods

### 2.1. Participants

This study reports a secondary data analysis from a larger study of parental adherence to the daily recommended treatment for their child’s CF. It took place at two accredited CF Centers, one a 523-bed academic pediatric medical center located in the US Midwest and the other a 290-bed academic pediatric medical center in the South. The institutional review boards at both institutions approved this study. Eligibility included having a child diagnosed with CF who was between three months and 13 years (the age at which parents are more than 70% responsible for their child’s treatment) [19], and the ability to read and speak English. For this secondary analysis, only participants who indicated they were married or were in a long-term relationship with a partner who resided with them (126 out of 141) were included. Of those participants who reported having a partner, 66 (52%) were members of a dyad in which both partners were participants in the study.

### 2.2. Procedure

Parents were informed of their eligibility for the primary study of spiritual beliefs and treatment adherence at their child’s quarterly outpatient visit and questions were answered. Either or both parents were eligible to participate. At site 1, verbal consent was obtained; at site 2, parents signed a written consent. Those who chose to enroll were provided a url address and asked to log on to the website and complete the study measures at their convenience using their own computers. If parents did not complete their data entry within one month, they received a follow-up contact offering to give them the url again. If both parents chose to participate, they each completed their own questionnaire responses separately. The questionnaire was set up via a REDCap Survey database [20] and included demographic questions, the Dyadic Adjustment Scale (DAS) [21], questionnaires concerning general coping, religious coping, sanctification of the body, spirituality, religiosity, congregational support, as well as constructs from the Theory of Reasoned Action (treatment utility, perceived behavioral norms, self-efficacy, and adherence intentions) [22]. Participants completed three Daily Phone Dairy (DPD) interviews within two weeks following completion of the questionnaires.

### 2.3. Measures

#### 2.3.1. Marital Adjustment

The Dyadic Adjustment Scale (DAS) was completed by participants who were married or had a life partner [21]. This well-used, reliable and valid 32-item scale has a possible range of 1–151. In addition to the total score, there are four subscales (Dyadic consensus, dyadic affection, dyadic satisfaction, and dyadic cohesion) which tap more specific domains of marital adjustment. The dyadic consensus subscale consists of 13 items to which respondents indicate the approximate extent of agreement or disagreement between themselves and their partner for each item. Responses are made on a six-point Likert-style scale ranging from 0 (always disagree) to 5 (always agree). An example item is, “Handling family finances.” The dyadic affection subscale is comprised of four items. Two inquire about extent of agreement with items using the same Likert-style scale as dyadic consensus (“demonstrations of affection” and “sex relations”). Two items as respondents to indicate if either item caused differences of opinions or were problems in the relationship in the past few weeks, and the response options are “yes” and “no”. These items are, “being too tired for sex” and “not showing love”. The dyadic satisfaction subscale is comprised of ten items. Seven are scored on a six-point Likert style scale ranging from 0 (all the time) to 5 (never); example items include, “how often do you discuss or have you considered divorce, separation, or terminating your relationship?” and “Do you confide in your mate?”). One item (do you kiss your mate?) is scored on a 5-point Likert-style scale ranging from 0 (never) to 4 (every day). One item asks respondents to indicate their degree of happiness in the relationship using a 7-point Likert-style scale ranging from 0 (extremely unhappy) and 6 (perfect). The final item in the dyadic satisfaction subscale asks respondents to choose one of six statements describing how they feel about the future of the relationship (0 = my relationship can never succeed and *there is no more I can do* to keep the relationship going”; 5 = I want desperately for my relationship to succeed, and *would go to almost any length* to see that it does). The dyadic cohesion subscale consists of five items to which respondents answer using a five-point Likert-style format ranging from 0 (none of them) to 4 (all of them); example items include, “Do you and your mate engage in outside interests together”, and, “laugh together”). The subscale score is the sum of the items comprising that subscale. Various authors have distinguished couples’ relationships as distressed or non-distressed based on cutoff values ranging from 92 to 107 on the total DAS score; the value of 100 was used as the cutoff in the present study [23].

#### 2.3.2. Spiritual Activities

Participants completed three Daily Phone Diary (DPD) calls, one of which took place Monday morning so as to include spiritual activities from those for whom Sunday is Sabbath. The DPD is a form of self-report using cued recall, in which participants recount all activities lasting more than five minutes in the previous 24 hours. In addition to naming the activity, they also report its duration, who was present during the activity, their mood (on a five point scale) and if the activity was recreational or instrumental. An initial prompt is given at the beginning of the interview to report certain activities of interest which may take less than five minutes, for example, taking supplemental enzymes or saying prayers before a meal. By asking about all activities, participants are less likely to give socially correct responses about the behavior of interest. Each activity is subsequently coded across three increasingly detailed levels of behavioral specificity. For the purposes of this study, only spiritual activities were included in the analyses; a copy of the spiritual activity codes is included as an appendix. Activity frequency and duration are averaged over the three DPD calls. All reported activities coded as spiritual were extracted from the DPD data and categorized as individual (neither partner nor any children were present), conjugal (partner was present but children were not), or familial (partner and at least one child were present). Average daily duration, in minutes, using reported beginning and ending times for the activity, as well as average daily frequency of each activity were calculated within each subject. Due to infrequent reporting of some activities and based on previous literature [24], all spiritual activities were subsequently collapsed and categorized as public (occurring in a public place of worship or spiritual group setting) or private (occurring within the home or other private sphere). Following these categorizations, we were able to analyze eleven total categories of activities: individual, conjugal, familial, public, private, and each combination of public/private and individual/conjugal/familial. The DPD has been widely used and has good psychometric properties [25–27].

#### 2.3.3. Disease Severity

Disease severity was measured by the number of pulmonary exacerbations requiring intravenous antibiotic therapy a child had over the twelve months prior to completing the questionnaire, and was obtained by chart review. The number of exacerbations requiring intravenous antibiotics is a frequent measure of disease severity in patients with CF [28,29].

### 2.4. Statistical Analyses

#### 2.4.1. Exploratory Data Analysis

Descriptive analyses, overall and by site, were performed with calculation of means, standard deviations, and medians for continuous measures, including duration and frequency of each spiritual activity, overall and subscale DAS scores, and number of pulmonary exacerbations in the year prior to study entry. A cubic transformation was used to achieve normality for overall and subscale distributions of DAS scores. DAS scores were dichotomized with those subjects with a score below 100 classified as having a distressed marriage or partnership and those at or above 100 have a non-distressed marriage [23]. Missing DAS subscales were imputed using hot deck imputation based upon those variables significantly differing between those with missing and non-missing DAS items [30,31]. Participants were classified by site and gender and sorted in serpentine fashion by variables found to differ between subjects with complete and incomplete DAS subscales. After sorting the observations, the missing DAS subscale value was borrowed from the most similar neighboring observation, resulting in a complete data set with a distribution of DAS scores very similar to the original distribution of non-missing observations. Categorical measures of patient characteristics are expressed as percentages. Differences between sites and parent gender with respect to continuous measures were examined with Student *t* tests.

#### 2.4.2. Inferential Analysis

Relationships (unadjusted) between DAS scores and spiritual activities were examined using Spearman correlation coefficients to test the first hypotheses. Differences between the participants who were above or below the clinical cutoff for distress with respect to frequency and duration of individual spiritual activities (the second hypothesis) were examined with Student *t* tests for continuous variables and Fisher’s exact test for categorical variables. To test the third hypothesis, multiple linear regressions were run in which the DAS scores were the response variable; the potential predictors were the frequency or duration of spiritual activities; number of pulmonary exacerbations within the last year was included as a covariate. The fourth hypothesis was tested as follows. The relationship between the probability of marital adjustment (measured by the dichotomized DAS score) and each type of spiritual activity (measured by the DPD) was examined with a generalized linear mixed model using the logit link function [32]. A random term for dyad effect was included in the model to account for the clustering arising from dyads in which both partners were participants in the study. In order to determine the amount of spiritual activity time which maintains marital adjustment, receive-operator characteristic (ROC) curves were generated. Two cut-off points for duration of spiritual activities were selected: first, the point maximizing both sensitivity and specificity; and second, Youden’s index (the point maximizing the quantity (sensitivity + specificity − 1) [33]. This index is commonly used as an overall measure of test accuracy. A value of 1 implies perfect prediction.

A *P* value less than 0.05 was considered statistically significant for key associations. Explanatory variables that met statistical significance at *P* < 0.10 were retained in regressions. In correlation analysis a *P* value less than 0.01 was considered significant due to the number of bivariate tests. Regression results for DAS scores have been back-transformed to their original scale. Results are expressed as mean (SD) or parameter estimate (95% CI) unless stated otherwise. Analyses were performed using SAS 9.3 (SAS Institute, Cary, NC).

## 3. Results

A total of 126 participants indicated they had a partner; of those, 88 also indicated at least one religious/spiritual activity during their Daily Phone Diary (DPD). Demographic data for the participants is presented in Table 1. To obtain DAS scores on all participants, the previously described imputation method was used to impute DAS subscales for 17 (13%) of participants who did not complete all subscales. Scores for the DAS and its subscales are presented in Table 1. Reliability analysis for the DAS was 0.92; Cronbach’s alpha values for subscales’ reliability were: dyadic consensus, 0.91; dyadic affection, 0.70; dyadic satisfaction, 0.55; dyadic cohesion, 0.85. No differences in mean DAS score by parent gender were found (female = 106 and male = 105; *p* = 0.45). Over half the 126 partnered parents were between 31 and 40 years old and the majority were mothers (n = 86; 68%). The children of these parents were relatively young (M = 5.6 years; SD = 4.1) and healthy, with less than one pulmonary exacerbation in the preceding year (M = 0.92; SD = 1.4). Parents spent the most time in individual spiritual activities (whether public or private) compared to time spent in conjugal or familial activities. Parents spent, on average, 15 minutes per day in private individual spiritual activities, 13 minutes on private conjugal and seven minutes on private familial activities. Parents spent longer average time on public spiritual activites than on private ones, with 110 minutes, on average in public spiritual activities, 10 minutes in conjugal and 70 minutes in familial public activities.

There was support for the first hypothesis, with significant correlations existing between frequency and duration of individual, conjugal and familial spiritual activities and scores on the DAS and subscales. Performing private spiritual activities more frequently and with longer duration were associated with higher marital adjustment (both 0.20, *p* = 0.027 and 0.029, respectively). Increasing the frequency and duration of private familial spiritual activities was associated with higher marital adjustment (0.20, *p* = 0.023, and 0.19, *p* = 0.031, respectively). The frequency and duration of private spiritual activities were significantly and positively correlated with marital adjustment. Frequency and duration of private individual and familial spiritual activities were significantly positively correlated with the dyad consensus subscale. Frequency and duration of private conjugal and familial spiritual activities were significantly positively correlated with the dyad satisfaction subscale. Public spiritual activities were not significantly correlated with scores on the DAS or any subscale. Lower marital adjustment was associated with having an increased number of pulmonary exacerbations (coefficient: −0.34, *p* = 0.0001). The DAS scale and all subscales were negatively correlated with the number of exacerbations in the past year.

The second hypothesis that significant differences would exist in the frequency and duration of spiritual activities for individuals with DAS scores below and above 100 was partially supported. There were significant differences in the frequency and duration of spiritual activities between those with DAS scores below 100 and above 100 (see Table 2). However, the significant differences were in individual spiritual activities rather than conjugal or familial. Disease severity, as measured by the number of pulmonary exacerbations in the preceding twelve months approached a significant difference (*p* = 0.055). The mean (SD) number of exacerbations found among parents in distressed marriages (DAS scores less than 100) was 1.4 (1.9), compared to 0.8 (1.2) in the group of parents in non-distressed marriages (DAS scores of 100 or more). Parental age category was significantly different between those with distressed versus non-distressed marriages with 70% of those parents in distressed marriages (DAS scores less than 100) aged 36 years or older while 70% of those with in non-distressed marriages (DAS score of at least 100) were 35 years or younger.

The third hypothesis, that the frequency of familial and conjugal spiritual activities would be significant factors in regression analysis of DAS scores after controlling for disease severity, was not supported. Only the number of exacerbations in the prior year and parent age met criterion for model inclusion. Parental age was a significant predictor in each of the regressions and was therefore controlled for in each model. Models which included conjugal spiritual frequency or duration are not reported due to the extremely low number of conjugal spiritual activities. Parent age was collected as a categorical variable as shown in Table 1; none of the categories achieved significance in the regression models (data not shown). However, both the number of exacerbations (*p* = 0.006–0.008) and parent age (*p* = 0.024–0.034) were significant in overall F-tests.

There was no support for the fourth hypothesis, that a cutoff value for duration of conjugal spiritual activities could be found which would have high sensitivity and specificity (probabilities > 0.80) and AUC (proportion > 0.90) for marital distress (classified as DAS < 100). However, increased duration of individual or private spiritual activities corresponded to a significant increase in the odds of having a DAS score of at least 100, (OR: 1.05, *p* = 0.022),while a greater duration of individual private spiritual activities was significantly associated with having a DAS of at least 100 (OR: 1.08, *p* = 0.04). These analyses included the random effect term to account for clustering due to having both partners’ data in the sample. ROC analyses showed that the cutoff value for total daily duration of spiritual activities that maximizes sensitivity and specificity was 12 minutes per day (AUC = 0.62). This level of daily duration did not correctly identify participants with marital distress (sensitivity: 0.46), but there was some evidence that it correctly identified participants who were not in marital distress (sensitivity: 0.77).

## 4. Conclusions

This paper presents analysis of individual, conjugal and familial spiritual behavior and their relationship to marital adjustment in a sample of parents of children with chronic illness. Religion and spirituality are important for many couples. Beliefs and practices predict marital outcomes and the attitudes partners have concerning their relationship [34]. Knapp and colleagues (2011) report that being married, being black non-Hispanic or “other” race were associated with higher levels of spirituality for parents of children receiving palliative care [35]. Overall, however, two-parent households had lower levels of spirituality than single-parent households. The institutional aspects of religion provide rituals which may assist in coping as well as instrumental forms of support to couples, especially those who have child with a chronic illness or special needs. Beliefs provide a framework within which to construct meaning of their experience, including reframing their experiences as parents [36,37]; viewing their God as a collaborator, interventionist, or other forms of control [9]. While religion and spirituality are normally associated with better marital outcomes, this appears to be the case only when beliefs and practices are operationalized; simple affiliation is not sufficient to obtain the marital benefits [34]. The present study presents preliminary data on the relationship between dyadic adjustment and religious and spiritual activities by individuals, couples and families in which a child has CF. Consistent with other studies, there was a positive relationship between frequency and duration of spiritual activities and marital adjustment. In fact, a relatively small amount of time in spiritual activities was associated with having a DAS score in the non-distressed range. At the same time, and also consistent with previous studies, child disease severity played a significant role in marital adjustment. Once disease severity was accounted for, the amount of time spent in spiritual activities ceased to have impact on marital adjustment scores. It may be that the beneficial effects of spiritual activities conferred on marital adjustment accrues to more typical marital situations (without the strain of a child with a life-shortening illness); the added strain overrides any positive impact spiritual activities have on the marriage. Alternatively, the burdensome treatment demands on parents of young children with CF may be sufficiently great that their focus is on their child’s health and survival, with relatively little time left to devote to maintaining a strong marriage.

A somewhat surprising finding from this study was that it was not conjugal or familial spiritual activity which related to marital adjustment but rather individual spiritual activity. There were very few conjugal spiritual activities overall, and thus insufficient data for any relationships to emerge in the analyses. This may reflect the strain placed on any marriage by children, who invariably reduce conjugal time generally, and conjugal time in public to a greater degree [38]. This strain would be exacerbated in a marriage in which a child has a chronic disease carrying a burdensome home treatment regimen. This is consistent with the imbalance between the frequency of private and public conjugal spiritual activities (nine private versus one public conjugal spiritual activity) in the present study. Parker suggests that pursuing individual activities contributes to marital adjustment by helping partners “remain interesting to each other”; Parker’s review of the literature suggest that it is the balance between time alone and time together which is important [39]. In this sample, it may be that the couples struck the balance between time alone and time together, and that a component of time alone was time devoted to individual spiritual activities. In the conceptual model by Pietromonaco and colleagues [5], seeking support is a relationship behavior that influences relationship outcomes. These results suggest that seeking support from the Divine, which represents a different dyadic relationship, nevertheless may influence relationship outcomes. Further, while family spiritual activities may enhance the family unit, they may have little impact on the functioning of the couple, especially for families with young children as in our sample. Family spiritual activities may be more focused on religious development of the children than enhancing the marital relationship. In the context of a chronic disease like CF, it is also possible that public family spiritual activities (such as attending congregational worship services) can lead to added strain on parents due to infection control concerns.

With the incidence of distressed marriages at about 25% in this sample, a proactive approach in which parents of recently-diagnosed children are informed of the potential new strain on their marriage may be beneficial. This would need to include making parents aware of what resources are available to them should this strain become significant. The relationship between marital adjustment and disease severity suggests that reassessment be related to milestones in disease severity. In the case of cystic fibrosis, that milestone could be pulmonary exacerbations requiring intravenous antibiotic therapy. Existing tools with good psychometric properties, such as the Psychosocial Assessment Tool (PAT) [40] include some marital questions, and could be a means of screening parents whose marital adjustment might warrant a deeper assessment or potential intervention. This is important because parents of chronically ill children face additional challenges compared to other married couples. They are more likely to have symptoms of depression and lower marital quality [41]. However, a study of parents of children with CF and parents of healthy children found no difference in marital satisfaction or depressive symptomatology [25]. Among children with developmental disabilities, higher marital quality predicted lower parenting stress and fewer symptoms of depression. Marital quality improved parenting efficacy for mothers only [42].

This study has the following limitations. By using CF as the disease exemplar, the sample demographic is limited to a predominately Caucasian sample. The use of two sites helped overcome some regional bias in religious demographics, but the sample was predominately Christian. The nature of the questionnaire did not provide information on exact age; therefore, information from age-related associations was limited to categories of age. Finally, the original study on which this exploratory data analysis was performed did not collect data on marriage duration. It is thus possible that it was not parental age which was a predictor of DAS scores in the regressions but rather duration of marriage, since presumably older parents were more likely to also have been married longer. This study also did not include parental socioeconomic status as a potential covariate. Nevertheless, important conclusions can be drawn. The DPD is a feasible tool to move the field forward by enabling investigators to study behaviors by dyads or families rather than individuals only. Marital adjustment is negatively impacted by child’s disease severity. Individual spiritual activities are positively associated with dyadic adjustment in this sample. Future research could build on this exploratory study by using a larger sample size and controlling for marriage duration, as well as including couples whose children have chronic diseases with significant treatment burdens other than CF.

## Figures and Tables

**Table 1:** Demographic characteristics of sample of parents and dyadic adjustment scale scores.

Characteristic		
Parent gender, N (%) Female		86 (68%)
Parent age (years), N (%)		
18–25		8 (6%)
26–30		21 (17%)
31–35		44 (35%)
36–40		28 (22%)
41–45		16 (13%)
>45		9 (7%)
Religious affiliation, N (%)		
Protestant		21 (17%)
Roman Catholic		23 (18%)
Non-denominational Christian		42 (33%)
Other		18 (14%)
None		22 (17%)
Child gender, N (%) Female		62 (50%)
Child age, mean (SD)		5.6 (4.1)
Pulmonary exacerbations in prior 12 mos, mean (SD)		0.92 (1.4)
DAS, mean (SD)		
Total		106 (18.7)
Dyadic cohesion		16.5 (4.6)
Dyadic affection		48.8 (8.9)
Dyadic consensus		8.6 (2.7)
Dyadic satisfaction		32.5 (5.0)
Religious spiritual activity duration in minutes, mean (SD)		
Private		
Individual (198 activities)		14.8 (16.4)
Conjugal (9 activities)		12.8 (13.3)
Familial (233 activities)		7.4 (9.8)
Public		
Individual (21 activities)		109.3 (188.4)
Conjugal (1 activity)		10.0 (0)
Familial (57 activities)		69.6 (57.4)

**Table 2:** Differences in mean frequency and duration of spiritual activities for participants with distressed and non-distressed marriages *.

	Distressed (DAS < 100) Mean (SD) N	Non-distressed (DAS ≥ 100) Mean (SD) N	P-value
Spiritual activity			
Individual			
Frequencies			
Total	0.4 (0.6) 30	0.8 (1.2) 96	0.044
Private	0.4 (0.6) 30	0.7 (1.0) 96	0.043
Duration			
Total	5.4 (13.8) 30	14.2 (29.8) 96	0.023
Private	2.7 (5.0) 30	11.6 (26) 96	0.011
Private			
Frequency	6.1 (8.3) 30	18.6 (29.7) 96	0.018
Duration	0.9 (1.0) 30	1.6 (1.6) 96	0.010
Total Duration	16.6 (33.9) 30	36.9 (53.9) 95	0.042
Child gender, N (%)			0.038
Female Male	21 (70%) 9 (30%)	46 (48%) 50 (52%)	
Parent age (years), N (%)			0.001
18–25	2 (6.7%)	6 (6.3%)	
26–30	1 (3.3%)	20 (20.8%)	
31–35	6 (20%)	38 (39.7%)	
36–40	9 (30%)	19 (19.9%)	
41–45	10 (33.3%)	6 (6.3%)	
>45	2 (6.7%)	7 (7.3%)	

Note:

* Only significant differences (*p* < 0.05) are reported for each comparison.

## Abbreviations

CF: cystic fibrosis
DAS: Dyadic Adjustment Scale
DPD: Daily Phone Diary

## Appendix

The following Activity Codes were used to code spiritual behaviors named in the Daily Phone Diary calls.

**Table:**

1	0	0	Spiritual Activities

1	1	0	Individual spiritual activities

1	1	1	praying
1	1	2	reading devotional material
1	1	3	watching religious programming on tv or internet
1	1	4	sacrament/other ritual (confession, healing)
1	1	5	visit cemetery by one’s self
1	1	6	talk with religious leader about spiritual issue(s)
1	1	7	talk with lay person about spiritual issue(s)
1	1	8	drive to/from spiritual activity
1	1	9	other individual spiritual activity

1	2	0	Group spiritual activities

1	2	1	family prayer (incl. grace at meals)
1	2	2	attend public worship
1	2	3	drive to/from or drop off youth group for child
1	2	4	drive to/from or drop off group spiritual activity (for self or family)
1	2	5	attend bible study
1	2	6	attend Sunday school/church school/Hebrew school/religious school
1	2	7	attend wedding, baptism, funeral, bat mitzvah, festival, etc.
1	2	8	watching religious programming on tv or internet
1	2	9	participate in 12-step recovery program (meeting or call sponsor)
1	2	10	attend social hour related to worship (coffee hour, breakfast)
1	2	11	visit cemetery with others
1	2	12	attend small group activity at place of worship
1	2	13	other group spiritual activity
1	2	14	reading religious/spiritual literature

1	3	0	Mind body spirit modalities

1	3	1	drive to/from mind body spirit modality
1	3	2	yoga
1	3	3	massage
1	3	4	chiropractic
1	3	5	art therapy
1	3	6	music therapy
1	3	7	aroma therapy (incl. candles)
1	3	8	acupuncture/acupressure
1	3	9	healing touch/reiki
1	3	10	other mind body spirit modality
1	3	11	writing (journal, diary, creative, poetry, etc.)
1	3	12	meditating
